# Differential Responses of Polyamines and Antioxidants to Drought in a Centipedegrass Mutant in Comparison to Its Wild Type Plants

**DOI:** 10.3389/fpls.2017.00792

**Published:** 2017-05-16

**Authors:** Mingxi Liu, Jingjing Chen, Zhenfei Guo, Shaoyun Lu

**Affiliations:** ^1^Department of Grassland Science, College of Agronomy, Hunan Agricultural UniversityChangsha, China; ^2^State Key Laboratory for Conservation and Utilization of Subtropical Agro-Bioresources, College of Life Sciences, Guangdong Engineering Research Center for Grassland Science, South China Agricultural UniversityGuangzhou, China; ^3^Key laboratory of Tropical Fruit Biology, Ministry of Agriculture, South Subtropical Crops Research Institute, Chinese Academy of Tropical Agricultural ScienceZhanjiang, China; ^4^College of Grassland Science, Nanjing Agricultural UniversityNanjing, China

**Keywords:** antioxidants, centipedegrass, drought, polyamines, turfgrass

## Abstract

Centipedegrass (*Eremochloa ophiuroides* [Munro] Hack.) is an important warm-season turfgrass species with low turf maintenance requirements. However, our knowledge on physiological adaptation of centipedegrass to drought stress is limited. Physiological responses to drought in a gamma-ray-induced mutant 22-1 as compared with two wild type (WT) lines were analyzed for understanding of drought tolerance mechanism of centipedegrass. The mutant showed an elevated drought tolerance with higher levels of relative water content, net photosynthetic rate (*A*) and stomatal conductance (*g*_s_) and lower levels of ion leakage and malondialdehyde (MDA) under drought stress as compared with WT plants. *A* showed significant correlation with *g*_s_ and MDA. Higher levels of antioxidant enzymes activities, non-enzyme antioxidants, and polyamines including putrescine (Put), spermidine (Spd), and spermine (Spm) were maintained in 22-1 than in WT plants. Superoxide dismutase (SOD), catalase (CAT), ascorbate-peroxidase (APX), and glutathione reductase (GR) activities and ascorbic acid (AsA) content were significantly correlated with both Put and Spd levels, and reduced glutathione level was correlated with Put during drought stress. Exogenous application of Put, Spd, and Spm increased drought tolerance and activities of SOD, CAT, APX, and GR in WT plants. The results suggest that higher levels of polyamines and antioxidant defense system are associated with the elevated drought tolerance in 22-1, which may improve protection on photosynthesis against drought induced oxidative damage.

## Introduction

Plant adaptation to drought involves various physiological responses. A desiccation-induced closure of stomata limits CO_2_ availability and reduces fixation through Bensen cycle and utilization of photo-generated reductant under drought, which leads to elevated production of reactive oxygen species (ROS), including superoxide radicals, hydrogen peroxide (H_2_O_2_) and hydroxyl radicals, by the water–water cycle (Asada, 1999). The accumulated ROS result in oxidative damages of photosynthetic apparatus when they could not be effectively scavenged (Foyer and Noctor, 2009). Antioxidant protection in plant cells confers to scavenge the accumulated ROS. Superoxide radicals are detoxified by superoxide dismutase (SOD), while H_2_O_2_ is scavenged by catalase (CAT) and the ascorbate–glutathione cycle including ascorbate peroxidase (APX), glutathione reductase (GR), ascorbate (AsA), and glutathione (GSH) (Gill and Tuteja, 2010).

Polyamines are important plant growth regulation substances, regulating plant growth, development and adaptation to environmental stresses (Minocha et al., 2014; Liu et al., 2015). Putrescine (Put), spermidine (Spd), and spermine (Spm) are three major constituents of polyamines in plants. Put is produced from arginine or ornithine, while Put and *S*-adenosylmethionine (SAM) are precursors for production of Spd and Spm (Alcázar et al., 2010; Gupta et al., 2013). Polyamines accumulate in rice (*Oryza sativa* L.) in response to drought and are associated with drought tolerance (Capell et al., 2004; Yang et al., 2007). Exogenous application of polyamines increased plant tolerance to drought or osmotic stress (Shi et al., 2010, 2013; Kotakis et al., 2014; Li et al., 2015a,b; Peng et al., 2016). Overexpression of *arginine decarboxylase* (*ADC*) and *S-adenosylmethionine decarboxylase* (*SAMDC*), the key genes for polyamine synthesis, resulted in elevated levels of Put, Spd, and Spm and enhanced drought tolerance in transgenic rice and tobacco plants (Waie and Rajam, 2003; Capell et al., 2004; Wi et al., 2014), while down-regulation of *SAMDC* gene decreased Spd and Spm levels and polyamines and led to reduced drought tolerance in transgenic rice plants (Chen et al., 2014).

Irrigation is one of the three primary cultures in turfgrass management, while drought is a major environmental factor limiting turfgrass growth and survival in semi-arid and arid regions across the world. Drought stress increases production of superoxide and H_2_O_2_, lipid peroxidation and some antioxidant enzyme activities in Kentucky bluegrass (*Poa pratensis* L.) (Bian and Jiang, 2009). Higher activities of APX and GR and lower levels of lipid peroxidation are observed in drought-tolerant cultivar ‘Midnight’ than in drought-sensitive cultivar ‘Brilliant’ of Kentucky bluegrass (Xu et al., 2011). Higher antioxidant enzyme capacity is associated with the higher drought tolerance in velvet bentgrass (*Agrostis canina* L.) than in colonial bentgrass (*Agrostis capillaris* L.) and creeping bentgrass (*Agrostis stolonifera* L.) (DaCosta and Huang, 2007). Association of antioxidant protection with drought tolerance has also been well documented in bermudagrass [*Cynodon dactylon* (L.) Pers.] (Lu et al., 2008; Chen et al., 2009) and hybrid bermudagrass (*Cynodon dactylon* ×*C. Transvaalensis*) (Lu et al., 2009). These studies reveal that drought tolerance in turfgrass species is associated with higher oxidative scavenging ability.

Centipedegrass is an important warm-season turfgrass species adapting to infertile soils and a range of climatic conditions in tropical and subtropical regions. Due to its low management requirements, good adaptation to poor soil fertility, and the current concerns for limited water resources, centipedegrass is increasingly used in residential lawns, recreational turf and soil conservation in subtropical to tropical zones and a grazing-purpose grass for low-input grassland systems in Japan (Hanna and Liu, 2003; Hirata et al., 2016). Centipedegrass was found to be able to transport heavy metals such as Pb from roots to shoots and leaves (Li et al., 2016), and thus it can be used for phytoremediation. Sequence-related amplified polymorphism (SRAP) (Milla-Lewis et al., 2012) and simple sequence repeat (SSR) markers (Harris-Shultz et al., 2012) have been used for genetic diversity in recent years. Genetic mapping and QTL analysis for seed yield, vegetative characters and cold tolerance were performed recently using two types of molecular markers, sequence-related amplification polymorphisms (SRAPs) and expressed sequence tags from wheat (*Triticum aestivum*) (Wang et al., 2014).

Despite its importance, limited breeding or selection efforts to improve centipedegrass have occurred, and very limited cultivars have been released due to its low diversity (Hanna and Liu, 2003). Gamma-ray radiation has been used to broaden the genetic and morphological variations in centipedegrass (Dickens et al., 1981; Hanna, 1995). A gamma-ray-induced mutant line with chilling tolerance has been selected (Lu et al., 2013). Higher levels of polyamines and antioxidants are associated with chilling tolerance in the chilling-tolerant mutant (Chen et al., 2013). Centipedegrass is considered to be drought tolerant (Hook et al., 1992; Huang et al., 1997); however, its physiological adaptation to drought is still unknown. A drought-tolerant mutant line 22-1 with red stems has been isolated from gamma-ray irradiated seeds of a commercial cultivar ‘Common’ through evaluations in the greenhouse and field tests (Lu et al., 2013). The objective of this study was to examine the differential physiological responses to drought between 22-1 and its WT plants with red stems. The results showed that higher levels of polyamines and antioxidant defense system were maintained in 22-1 than in WT plants under drought stress conditions, while the higher activities of antioxidant enzymes were associated with polyamine levels. The observation is important for breeding of centipedegrass for improved drought tolerance using transgenics in the future.

## Materials and Methods

### Plant Materials, Growth Conditions, and Treatments

The mutant line 22-1 with red stems were investigated in this study, while two individual seed-germinated plants of the wild type Common with red-stemmed stolons, ZC1 and ZC2, were chosen randomly and used as control plants. Two wild type lines were used because centipedegrass has a high level of self-incompatibility and seed is largely formed by cross-pollination (Hanna and Liu, 2003), thus each individual plant derived from seed are a population of genotypes. The plugs with similar size of 22-1 along with the wild type control plants were transplanted to plastic pots (10 cm in diameter and 15 cm in depth) containing a mixture of peat and perlite (3:1, v/v). Plants were allowed for growing at least 60 days in a greenhouse at temperature of 20 to 30°C under natural light, with routine management by daily irrigation, biweekly mowing at 4 cm and biweekly fertilizing with 0.3% solution of 15N–6.6P–12.5K fertilizer. For drought treatment, plants were fully irrigated, followed by withholding irrigation for 7 days in the greenhouse. Three pots were used as replications for each plant line, which were arranged as a completely randomized design. For treatment with polyamines, plants were irrigated with 50 ml of 0.1 or 0.5 mM Put, Spd, or Spm solution per pot, followed by sampling for measurements of antioxidant enzyme activity 2 days after treatment or by withholding irrigation as described above. The third leaf from the top was collected for measurements.

### Determinations of Relative Water Content (RWC) and Ion Leakage

Relative water content (RWC) and ion leakage were determined from the leaves of pot plants as previously described (Lu et al., 2008). For measurement of RWC, fresh leaves were weighed (W_f_) and immersed in water overnight until the weight of the leaves was constant. The water-saturated leaves were weighed (W_S_) and then dried for 24 h at 80°C for determinations of the dry weight (W_d_). RWC was calculated by the formula: RWC = (W_f_–W_d_)/(W_S_–W_d_) × 100. For measurement of ion leakage, leaf samples were immersed in 10 ml of distilled water overnight. The conductivity of the solution (*C*_1_) was measured using a conductivity meter (Model DDS-11A, Shanghai Leici Instrument Inc., Shanghai, China). After the samples were heated by incubating in boiling water (100°C) for 20 min and cooled to room temperature, the conductivity of killed tissues (*C*_2_) was again measured. Ion leakage was calculated as (*C*_1_/*C*_2_) × 100.

### Determinations of Malondialdehyde (MDA) and Enzyme Activities

Fresh leaves (0.5 g) were ground in a mortar with pestle in 5 ml of 50 mM phosphate buffer (pH 7.8) at 4°C. The homogenate was centrifuged at 15,000 × *g* for 15 min. The supernatant was recovered for determinations of malondialdehyde (MDA) and activities of SOD, CAT, and GR as previously described (Guo et al., 2006). APX was extracted by grinding leaves (0.5 g) in a mortar with pestle in 5 ml of 50 mM phosphate buffer (pH 7.0, containing 1 mM ascorbic acid and 1 mM EDTA) at 4°C, followed by centrifugation as above. The 3-ml reaction solution of SOD contained 13 μM methionine, 63 μM ρ-nitro blue tetrazolium chloride, 1.3 μM riboflavin, 50 mM phosphate buffer (pH 7.8), and enzyme extract. The reaction solution was incubated for 10 min under fluorescent light with 80 μmol m^-2^ s^-1^. Absorbance was determined at 560 nm with a spectrophotometer (Model UV-2010, Hitachi, Japan). The 3-ml reaction solution of CAT contained 15 mM H_2_O_2_, 50 mM phosphate buffer (pH 7.0), and 50 μl of enzyme extracts. The reaction was initiated by adding enzyme extracts. The decrease of absorbance of H_2_O_2_ within 1 min at 240 nm was recorded. The 1-ml reaction mixture of GR contained 0.1 mm NADPH, 40 mM Tricine-NaOH (pH 7.8), and 0.2 ml of enzyme extracts. The reaction was initiated by the addition of 0.5 mm oxidized GSH and the rate of NADPH oxidation was monitored at 340 nm within 1 min. The 3-ml reaction solution of APX contained 50 mM phosphate buffer (pH 7.0), 0.5 mM AsA, 0.1 mM H_2_O_2_, and 0.1 ml enzyme extracts. APX activity was calculated by following the decrease in absorbance of AsA (extinction coefficient 2.8 mM^-1^ cm^-1^) within 1 min at 290 nm. The concentration of MDA was calculated using a coefficient of absorbance of 155 mM^-1^ cm^-1^. One unit of SOD activity was defined as the amount of enzyme required for inhibition of photochemical reduction of ρ-nitro blue tetrazolium chloride (NBT) by 50%. One unit of CAT and APX was defined as the amount of enzyme required for catalyzing the conversion of one μmol H_2_O_2_ (extinction coefficient 0.0394 mM^-1^ cm^-1^) or AsA (extinction coefficient 2.8 mM^-1^ cm^-1^) within 1 min. One unit of GR was defined as the amount of enzyme required for catalyzing oxidation of one μmol NADPH (extinction coefficient 6.22 mM^-1^⋅cm^-1^) within 1 min.

### Determinations of AsA and GSH

Fresh leaves (1 g) were ground in a mortar with pestle in 5 ml of 5% trichloroacetic acid at 4°C. The homogenates were centrifuged at 13,000 × *g* for 15 min. AsA and GSH were determined as previously described (Zhou et al., 2005). AsA was measured using bipyridyl, while GSH was determined using 5,5′-Dithio-bis (2-nitrobenzoic acid) (DTNB). For measurement of AsA, supernatants (0.4 ml) was combined with 0.2 ml of NaH_2_PO_4_ buffer (150 mM, pH7.4). To this mixture 0.4 ml of 10% (w/v) trichloroacetic acid, 0.4 ml of 44% (v/v) H_3_PO_4_, 0.4 ml of 4% (w/v) bipyridyl in 70% (v/v) ethanol, and 0.2 ml of 3% (w/v) FeCl_3_ was added. After vortex, the mixture was incubated at 37°C for 60 min and the absorbance at 525 nm was recorded. For measurement of GSH, supernatant (0.2 ml) was added to 2.6 mL of 150 m*M* NaH_2_PO_4_ (pH 7.4). 0.2 ml of 5,5-dithio-bis(2-nitrobenzoic) (DTNB) (75.3 mg of DTNB was dissolved in 30 ml of 100 mM phosphate buffer, pH 6.8) was then added. The mixture was incubated at 30°C for 5 min. Absorbance was determined at 412 nm. Concentrations of AsA and GSH were calculated by comparison to a standard curve.

### Determination of Net Photosynthetic Rate

Net photosynthetic rate (*A*) and stomatal conductance (*g*_s_) were measured as described previously (Chen et al., 2013), using a LI-6400P Portable Photosynthesis System (LI-COR Inc., Lincoln, NE, USA) according to the manufacture’s instructions. The measurement was conducted after 10 min equilibration to achieve steady state conditions in the leaf chamber: photosynthetic active radiation (PAR) was maintained at 1200 μmol⋅m^-2^⋅s^-1^, temperature was controlled at 25°C and CO_2_ concentration at 400 μmol mol^-1^ at 70% relative humidity.

### Determination of Polyamine Concentrations

Polyamines were extracted from leaves (0.5 g) with 4 ml of freshly prepared 5% (v/v) perchloric acid and extracted at 4°C, followed by centrifugation at 15,000 *g* for 30 min. The supernatants were used for detection of free polyamines using high-performance liquid chromatography (HPLC) as previously described (Chen et al., 2013; Guo et al., 2014). Aliquots (0.5 ml) were benzoylated as described by Flores and Galston (1982). The benzoylated polyamines were re-suspended in 1 ml of mobile phase solution and filtered (4.5 μm filter) before HPLC analysis. Twenty microliter of sample was injected into a Waters chromatographic system (Waters, Mildford, MA, USA), supplied with a C18 column (Dalian Elite Analytical Instruments Co., Ltd., Dalian, China, 4.6 mm × 250 mm). The mobile phase was 64% methanol in an isocratic elution, at a flow rate of 0.7 ml min^-1^ and ambient temperature. Identification and quantification of Put, Spd, and Spm in each sample were achieved by comparing each peak retention time and peak area with the standard polyamines, being detected at 254 nm using a 2487 dual UV detector (Waters, Milford, MA, USA). Put, Spd, and Spm contents were calculated using standard curves with commercial standards and a correction for recovery after the extraction procedure. Three pots of plants were used for measurement as replications.

### Statistical Analysis

The experiments were arranged in a completely randomized design with three pots of plants as replicates. For measurements of *A* and *g*_s_, five leaves in each pot were randomly chosen and used for assay independently. For all the biochemical and physiological measurements, a pooled material from several different plants in each pot was randomly collected and used for assay. Significance of differences in the various parameters was assessed by One-way ANOVA (*p* < 0.05) using an SPSS program (SPSS Inc, Chicago, IL, USA).

## Results

### The Mutant 22-1 had Elevated Drought Tolerance Compared with Its WT Plants

**FIGURE 1:**
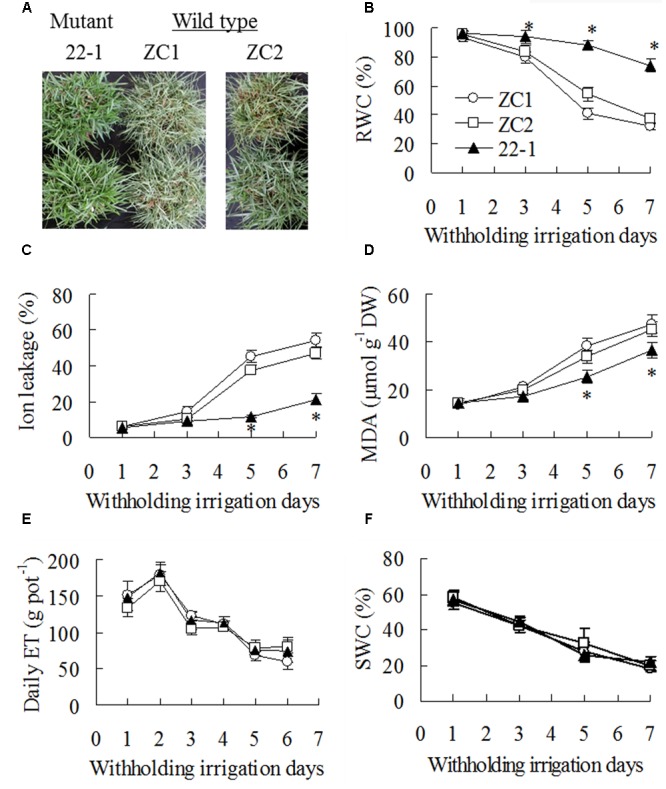
**Assessment of drought tolerance in the mutant 22-1 in comparison with two wild type plant lines (ZC1, ZC2).** Photography was taken after 7 days of withholding irrigation **(A)**. Relative water content (RWC, **B**), ion leakage **(C)**, malondialdehyde (MDA, **D**), daily evaporation (ET, **E**), and soil water content (SWC, **F**) were measured at the dates as indicated in the figures. Means of three pots of plant samples and the standard errors are presented; an asterisk indicates significant difference between 22-1 and the wild types at *P* < 0.05 at a given time.

Compared to being serious wilting in WT plants, 22-1 maintained turgid after 7 days of withholding irrigation (**Figure 1A**). RWC was decreased in all plants with withholding irrigation time, and higher RWC was maintained in 22-1 than in WT plants (**Figure 1B**). Similarly, ion leakage and MDA showed an increase after drought treatment, while lower levels were maintained in 22-1 than in WT plants at 5 and 7 days (**Figures 1C,D**). However, daily evapotranspiration (ET) showed no difference between 22-1 and WT plants during withholding irrigation (**Figure 1E**), which led to no difference in soil water content (SWC) among all tested plants (**Figure 1F**).

**FIGURE 2:**
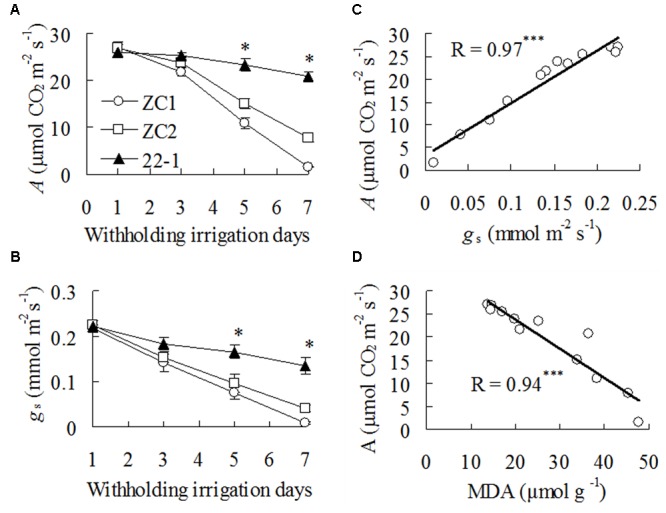
**Net photosynthetic rate *(A)* and stomatal conductance (*g*_s_) in the mutant 22-1 and the wild type plants (ZC1, ZC2) and correlation of *A* with *g*_s_ and MDA during drought stress. *A* (A)** and *g*_s_ **(B)** were measured at the dates as indicated in the figures. Correlation of *A* with *g*_s_ **(C)** and MDA **(D)** and during withholding irrigation were analyzed using linear regression. Means of three pots of plant samples and the standard errors are presented; the asterisks ^∗∗∗^ indicate significant difference between 22-1 and the wild types at *P* < 0.001.

Net photosynthetic rate (*A*) and *g*_s_ showed a slight decline after 3 days of withholding irrigation in WT plants (**Figures 2A,B**), but *A* was not altered in 22-1 (**Figure 2A**). *A* and *g*_s_ were greatly decreased at 5 to 7 days, and higher levels were maintained in 22-1 than in WT plants (**Figures 2A,B**). In addition, *A* showed a highly positive correlation with *g*_s_ and a negative correlation with MDA during drought treatment (**Figures 2C,D**).

### Higher Levels of Antioxidants Maintained in the Mutant than in WT under Drought Conditions

**FIGURE 3:**
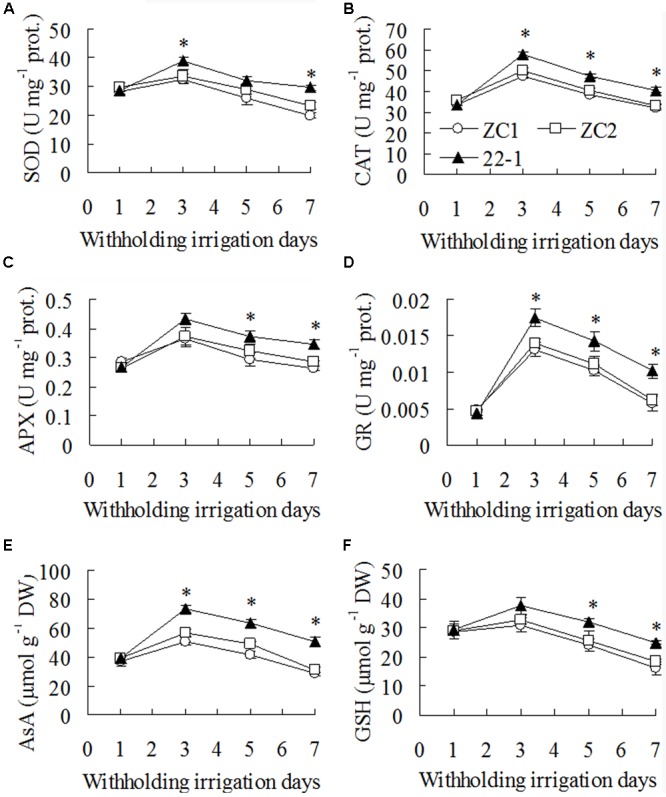
**Superoxide dismutase (SOD, **A**)**, ascorbate-peroxidase (APX, **B**), catalase (CAT, **C**) and glutathione (GSH) reductase (GR, **D**) activities and ascorbic acid (AsA, **E**) and reduced GSH **(F)** contents in the mutant 22-1 in comparison with two wild type plant lines (ZC1, ZC2) in response to drought stress. Means of three independent plant samples and the standard errors are presented; an asterisk indicates significant difference between 22-1 and the wild types at *P* < 0.05 at a given time.

Activities of SOD, CAT, APX, and GR showed no difference between 22-1 and the wild type plants before drought treatment. They were increased after 3 days of withholding irrigation, followed by a continuous decrease, and higher activities were maintained in 22-1 than in the wild type plants (**Figures 3A–D**). For example, activities of SOD, CAT, APX, and GR were higher by 17 to 36%, 18 to 25%, 18 to 26%, and 29 to 72%, respectively, in 22-1 than the means of two WT plants after 3 to 7 days of withholding irrigation (**Figures 3A–D**). AsA and GSH showed the similar pattern in response to drought treatment. They were increased after 3 days of withholding irrigation, followed by a continuous decrease. AsA and GSH were 36 to 68% and 18 to 45% higher in 22-1 than the means of two WT plants, respectively, after 3 to 7 days of withholding irrigation (**Figures 3E,F**).

### Higher Levels of Polyamines in the Mutant than in WT under Drought Conditions

**FIGURE 4:**
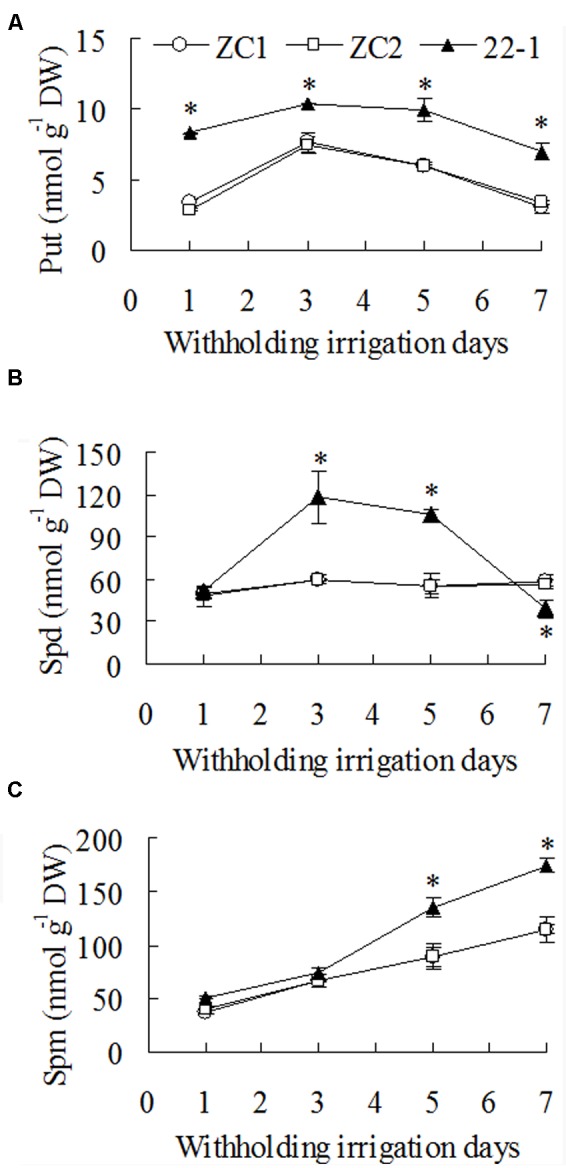
**Putrescine (Put, A)**, spermidine (Spd, **B**), and spermine (Spm, **C**) levels in the mutant 22-1 in comparison with two wild type plant lines (ZC1, ZC2) in response to drought stress. Means of three independent plant samples and the standard errors are presented; an asterisk indicates significant difference between 22-1 and the wild types at *P* < 0.05 at a given time.

A significantly higher level of Put (1.7-fold) was observed in 22-1 than in WT plants before plants were exposed to drought stress treatment. Put level was increased after 3 days of withholding irrigation, followed by a decrease at 5 and 7 days, and higher levels were maintained in 22-1 than in WT plants. There was 37 to 115% higher level of Put in 22-1 than the means of two WT plants from 3 to 7 days after withholding irrigation (**Figure 4A**). Spd level showed no change in WT plants during withholding irrigation, while it was increased significantly in 22-1 after 3 to 5 days of withholding irrigation, followed by a decrease at 7 days (**Figure 4B**). Spd level was 90 to 96% higher in 22-1 than the means of two WT plants from 3 to 5 days after withholding irrigation (**Figure 4B**). Spm level was continuously increased with withholding irrigation time, and significantly higher levels was observed in 22-1 than in WT plants at 5 and 7 days (**Figure 4C**). Spm level was 52% higher in 22-1 than the means of two WT plants from 5 to 7days after withholding irrigation (**Figure 4C**).

Correlation between polyamine levels and antioxidant enzyme activities in both 22-1 and WT plants during withholding irrigation was analyzed. Both Put and Spd levels were significantly correlated with SOD, CAT, APX, and GR activities and AsA and GSH contents, and Put level was correlated with GSH, while Spm level showed no correlation with any antioxidant (data not shown).

### Exogenous Polyamines Increased Drought Tolerance and Antioxidant Enzyme Activities

**FIGURE 5:**
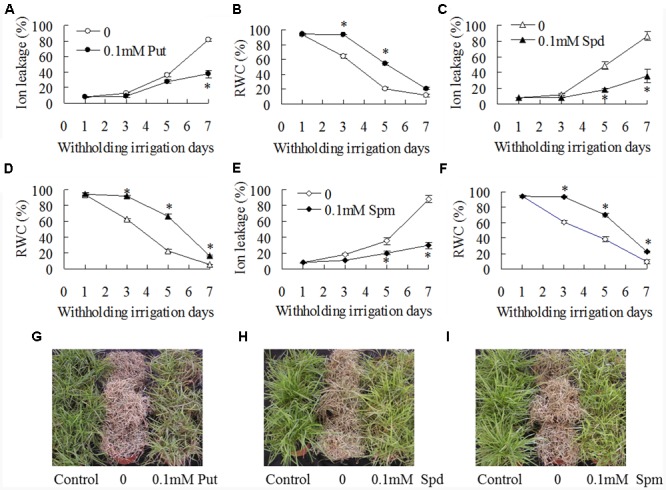
**Effects of exogenous putrescine (Put), spermidine (Spd), and spermine (Spm) on drought tolerance.** The wild type (ZC1) plants were irrigated with 50 ml of 0.1 mM Put, Spd, or Spm solution per pot, followed by withholding irrigation. Ion leakage **(A,C,E)** and relative water content **(B,D,F)** were measured at the dates as indicated in the figures. Photography was taken after one week of recovery by re-watering plants post withholding irrigation **(G–I)**. Means of three independent plant samples and the standard errors are presented; an asterisk indicates significant difference between treatment with polyamines and untreated control at *P* < 0.05 at a given time.

**FIGURE 6:**
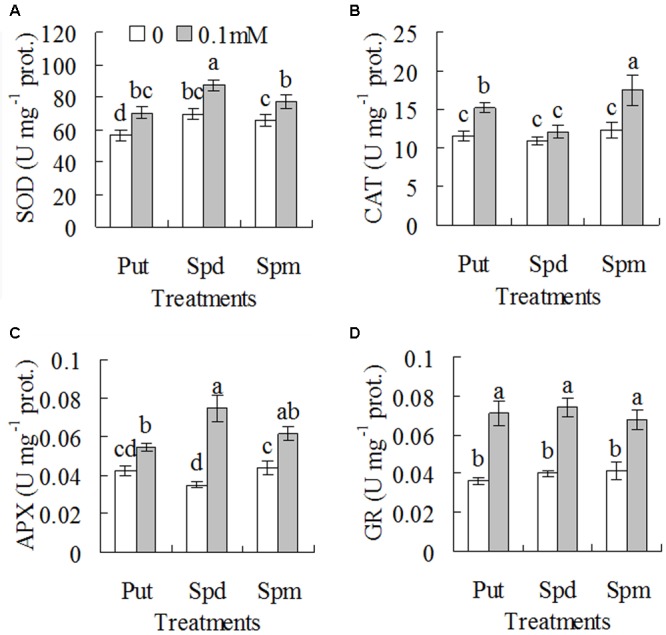
**Activities of superoxide dismutase (A)**, catalase **(B)**, ascorbate-peroxidase (APX, **C**), and GSH reductase **(D)** in the wild type plants (ZC1) as affected by exogenous putrescine (Put), spermidine (Spd), and spermine (Spm). Means of three independent plant samples and the standard errors are presented; the same letter indicates no significant difference at *P* < 0.05 within the same polyamine treatment.

Effects of exogenous polyamines on drought tolerance and antioxidant enzyme activities in WT plants were further examined. Ion leakage and RWC were measured for assessment of drought tolerance. Ion leakage and RWC were increased with withholding irrigation days, while significantly lower ion leakage and higher RWC were observed in 0.1 mM Put, Spd, or Spm treated plants than in untreated controls (**Figures 5A–F**). For example, ion leakage was lower by 22, 63, and 42%, respectively, in the plants treated with Put, Spd, and Spm than that in untreated controls after 5 days of withholding irrigation, and RWC was higher by 160, 190, and 80%, respectively (**Figures 5A–F**). Untreated plants could not be recovered and became dead after re-irrigation after 8 days of withholding irrigation, while most of the plants treated with 0.1 mM Put, Spd, or Spm could survive (**Figures 5G–I**). In addition, significantly higher activities of SOD, CAT, APX, and GR were observed in 0.1 mM Put, Spd, or Spm treated plants than in untreated plants (**Figures 6A–D**). SOD activity was increased by 18 to 25% after treatments with Put, Spd, and Spm; CAT was increased by 11 to 43%; APX activity was increased by 29 to 114%; GR activity was 65 to 96% (**Figures 6A–D**).

## Discussion

An enhanced drought tolerance in mutant 22-1 has been shown in our previous selections on the field and greenhouse evaluations (Lu et al., 2013). Higher RWC and lower ion leakage were maintained in 22-1 than in WT plants during drought stress demonstrated the enhanced drought tolerance in 22-1. MDA is a product of membrane-lipid peroxidation and an index of oxidative injury of plants. Lower levels were observed in 22-1 than in WT plants during drought stress, indicating that less oxidative injury was occurred in 22-1 under drought stress. Photosynthesis is inhibited by water deficits, via decreased CO_2_ diffusion to the chloroplast and metabolic constraints. Stomata limitation is generally considered the main cause for decreased photosynthesis under mild to moderate water stress conditions (Grassi and Magnani, 2005; Chaves et al., 2009). *A* and *g*_s_ decreased in response to drought in both bermudagrass and Kentucky bluegrass, with higher *A* in drought-tolerant cultivar than in sensitive cultivar (Hu et al., 2009, 2010). The similar results were also observed in this study, and *A* showed positive correlation with *g*_s_, indicating that stomata limitation was associated with the decreased *A*. In addition, the decreased *A* was highly correlated with the increased MDA, and higher *A* was maintained in 22-1 than in WT plants. The results indicated that the higher photosynthesis capacity in 22-1 was also associated with the less oxidative injury.

Antioxidant defense system functions to protect plants against abiotic stress caused oxidative damages through scavenging of ROS. ROS is generated and accumulated under drought stress due to imbalance between production and utilization of photo-generated reductant, as a result of water desiccation-induced stomata closure which limits CO_2_ availability and reduces fixation through Bensen cycle. APX, CAT, and GR activities and AsA and GSH contents were increased after 3 days of withholding irrigation, when *g*_s_ was decreased which reflected a closure of stomata. The enhanced antioxidant defense system may reflect an adaptation of centipedegrass to drought to cope with an accumulated ROS. Higher levels of antioxidants were maintained in 22-1 than in WT plants, which may contribute for less accumulation of ROS under drought and lead to lower levels of ion leakage and MDA and higher levels of *A* in 22-1 as compared with WT plants, even though antioxidant levels were decreased in all plants with the drought stress time after 5 days of withholding irrigation. Higher activities of antioxidant enzymes are observed in drought-tolerant cultivars than in sensitive ones of turfgrass species and other crops under drought conditions (Jiang and Huang, 2001; Guo et al., 2006; DaCosta and Huang, 2007; Lu et al., 2008, 2009; Chen et al., 2009). The results indicated that maintenance of higher antioxidant levels during drought stress plays an important role in drought resistance of centipedegrass.

Polyamines are involved in plant adaptation to abiotic stresses. Although the molecular mechanism of polyamines is still not understood, improved tolerance to drought stress was observed in transgenic plants up-regulating polyamine synthesis (Waie and Rajam, 2003; Capell et al., 2004; Wi et al., 2014) or by exogenous application of polyamines (Shi et al., 2010, 2013; Kotakis et al., 2014; Li et al., 2015a,b; Peng et al., 2016), while down-regulation of polyamine synthesis led to reduced drought tolerance in transgenic plants (Chen et al., 2014). Higher levels of Put, Spd, and Spm were observed in 22-1 than in WT plants during drought stress, and treatments with exogenous Put, Spd, or Spm increased drought tolerance of WT plants of centipedegrass. Nevertheless, our data indicated that the higher levels of polyamines in 22-1 are associated with the elevated drought tolerance. Polyamines could be catalyzed by PAO to produce H_2_O_2_ in apoplast. The accumulated H_2_O_2_ as a result of polyamine oxidation leads to induced expression of antioxidant enzyme encoding genes in tobacco plants (Guo et al., 2014). In addition, exogenous Spd induced H_2_O_2_ burst as a result of induced activity of NADPH oxidase in white clover (Li et al., 2015b). Both Put and Spd levels showed significant correlation with activities of SOD, CAT, APX, and GR and contents of AsA and GSH contents, and Put level was correlated with GSH in this study, indicating that antioxidant system might be regulated by polyamines in centipedegrass. This was confirmed by the observation that exogenous application of Put, Spd, or Spm resulted in enhanced activities of SOD, CAT, APX, and GR as well as drought tolerance. Moreover, treatment with polyamines increased activities of antioxidant enzymes and reduces oxidative damages in chickpea (*Cicer arietinum* L.) (Nayyar and Chander, 2004), *Brassica juncea* (Verma and Mishra, 2005), and white clover (Li et al., 2015b; Peng et al., 2016). Spd is involved in osmotic stress-induced transient rice of H_2_O_2_, Ca^2+^, and NO signal molecules which activates antioxidant enzyme activities and gene expression (Li et al., 2015b; Peng et al., 2016). Exogenous polyamines increase tolerance to drought and salt stresses in bermudagrass (*Cynodondactylon*) with significantly increased the abundance of antioxidant enzymes and several other stress-related proteins (Shi et al., 2013), while down-regulation of polyamine synthesis resulted in reduced antioxidant enzyme activities and drought tolerance in transgenic rice (Chen et al., 2014). Our results suggest that the differential accumulation of Put, Spd, and Spm during drought, which led to the differential antioxidant defense capacity, was associated with the differential drought tolerance between 22-1 and WT plants. Our data provide a clue with up-regulation of polyamine biosynthesis to improve drought tolerance in centipedegrass by transgenics in future.

## Conclusion

The enhanced drought tolerance in 22-1 was associated with higher levels of antioxidants as compared with WT plants as a result of higher levels of Put and Spm during drought or Spd under mild and moderate drought stress, which resulted in better protection of photosynthesis apparatus against drought stress induced oxidative damage.

## Author Contributions

ML and JC carried out the experiment and participated in data analysis. ZG and SL designed the experiment, analyzed the data, and drafted the manuscript. All the authors have revised this manuscript critically before the submission and agreed with all aspects of the work.

## Conflict of Interest Statement

The authors declare that the research was conducted in the absence of any commercial or financial relationships that could be construed as a potential conflict of interest.
